# A Novel Coating of Orthodontic Archwires with Chlorhexidine Hexametaphosphate Nanoparticles

**DOI:** 10.1155/2023/9981603

**Published:** 2023-03-15

**Authors:** Zahraa Mohammed Al-Fadhily, Mehdi Abdul-Hadi

**Affiliations:** ^1^Department of Pedodontics, Orthodontics and Preventive Dentistry, College of Dentistry, University of Kufa, Najaf, Iraq; ^2^Department of Orthodontics, College of Dentistry, University of Baghdad, Baghdad, Iraq

## Abstract

**Materials and Methods:**

A solution of CHX-HMP nanoparticles with an overall concentration of 5 mM for both CHX and HMP was prepared, characterized (using atomic force microscope and Fourier transformation infrared spectroscopy), and used to coat orthodontic stainless steel (SSW) and NiTi archwires (NiTiW). The coated segments were characterized (using scanning electron microscopy SEM with energy dispersive X-ray spectrometry and field emission SEM) and subjected to the elusion assessment.

**Results:**

After having their composition validated, the average size of the CHX-HMP NPs was assessed to be 51.21 nm, and the analysis revealed that the particles had both chlorine and phosphorus. After 30 minutes in the coating solution, NPs deposited on the surface of the SSW and NiTiW. A continuous release of soluble CHX in artificial saliva was detected from both SSW and NiTiW as long as the experiment lasted for 28 days without reaching a plateau. However, the release from coated NiTiW was significantly more than coated SSW at 7, 14, and 28 days. While at day 21, the release from coated SSW was slightly greater than that from the coated NiTiW.

**Conclusion:**

Orthodontic stainless steel and NiTi archwires can be successfully coated with CHX-HMP NPs and give sustained release of CHX along the examined period.

## 1. Introduction

Orthodontic therapy aims to improve the self-image of patients by giving more pleasant and attractive smiles, besides enhancing oral functions [1, 2]. Fixed orthodontic appliances are the treatment of choice for the vast majority of malocclusions [3]. However, the oral microbiome may alter as a result of orthodontic treatment [4, 5]. Components of a fixed appliance such as brackets, bands, elastics, axillaries, and extra composite material around the brackets have irregular surfaces and intricate undercut designs. Therefore, they make more places for plaque to stick to the smooth surfaces of the teeth, which are usually less affected by cavities [6]. White spot lesions or carious demineralization are common unintended consequences of orthodontic therapy. Many diverse scientific studies had concluded that orthodontic procedures may considerably raise the risk of the presence of white spot lesions [7–9].

According to research studies, white spot lesions may progress rapidly in the first few weeks of orthodontic treatment [10, 11]. Additionally, the rate of demineralization around fixed orthodontic appliances is higher than the typical rate of carious lesion development, as evidenced by the onset of enamel demineralization primarily within the first six months of starting the orthodontic treatment. The prevalence may be more than 46% within 12 months of fixed appliance bonding [10]. A longitudinal study conducted by Alidan and Alrawi in 2017 found that 74.61% of all teeth were affected by white spot lesions six months after the beginning of the treatment [12] As an unpleasant, unsightly, and sometimes irreversible lesion needs restoration, white spot lesions prevention is a crucial concern for orthodontists [13].

Furthermore, the increase in the oral microbial community together with significant variations in subgingival microbiomes and a higher level of bacterial resistance associated with fixed appliance intervention led to a rise in the incidence of localized gingivitis/mild periodontitis [14]. From the start of orthodontic treatment, the plaque index and gingival index began to elevate [15]. Zanatta et al. stated that orthodontic treatment was linked to an increase in gingivitis in 56.8% of adolescent patients and 34.4% of adult patients [16].

It is widely accepted that good oral hygiene is a significant preemptive measure that helps patients' gingival and periodontal tissues stay healthy and recover after treatment. Moreover, lesions and cavities can be avoided by eliminating bacterial plaques using mechanical methods, mouthwashes, and fluoride applications [17]. However, patient compliance is crucial to the success of this prophylactic strategy [18]. Most patients having a fixed orthodontic appliance installed are associated with a significant decline in oral health and cleanliness, which is typically attributed to the patient's discomfort as the teeth adjust to their new position and difficulty in keeping good oral hygiene with the presence of multiple irregular surfaces [19]. Therefore, coating orthodontic devices with antimicrobial components, like specific nanoparticles, can be investigated as an alternative technique for avoiding adherence and microbial colonization [20].

Nanoparticle materials are specified as “materials exhibiting outstanding properties in the size range of 1 to 100 nm” [21]. When materials attain nanometric dimensions, their properties, such as increased toughness, larger active surface area, greater chemical, and biological activity, change. The antimicrobial activity is boosted due to the particle's small size and a high surface-to-volume ratio [22, 23]. This improves the material's biocompatibility and interaction with the microbial membrane, hence accelerating the creations of new devices and technologies for application in the physics, biology, biomedicine, and pharmaceutical industries [23–25].

Chlorhexidine is a cationic biguanide that own obvious spectrum antimicrobial properties, making it efficient against a vast range of bacteria and yeasts and does not encourage the development of bacterial resistance [26]. A novel salt of CHX-hexametaphosphate nanoparticles (CHX-HMP NPs) are described as a substance when subjected to an aqueous environment that gives sustained release of the CHX constituent. Specimens of glass, titanium [26], dental implants [27], elastomeric ligatures [28], elastomeric chains [29], and orthodontic miniscrew were effectively coated with CHX-HMP NPs that gave a sustained release of soluble CHX for an extended time period [30].

To the researchers' knowledge, no previous research had been conducted concerning coating orthodontic archwires with CHX-HMP NPs. This study aimed to test the capability of coating orthodontic archwires with CHX-HMP NPs, evaluate the elusion of CHX from coated archwires, and how long CHX elusion may persist.

The null hypothesis for this study was that the CHX-HMP NPs neither have the capability of coating orthodontic archwires nor allow the release of CHX for a long time.

## 2. Materials and Methods

### 2.1. Materials and Reagents

The study sample includes specimens consisting of 0.019 × 0.025-inch stainless steel and NiTi orthodontic upper archwires (Dentaurum GmbH & Co. KG, Ispringen, Germany). A chlorhexidine digluconate solution and sodium hexametaphosphate crystalline (Sigma Aldrich, Germany) were employed for nanoparticle synthesis.

### 2.2. Overview

**Figure 1:**
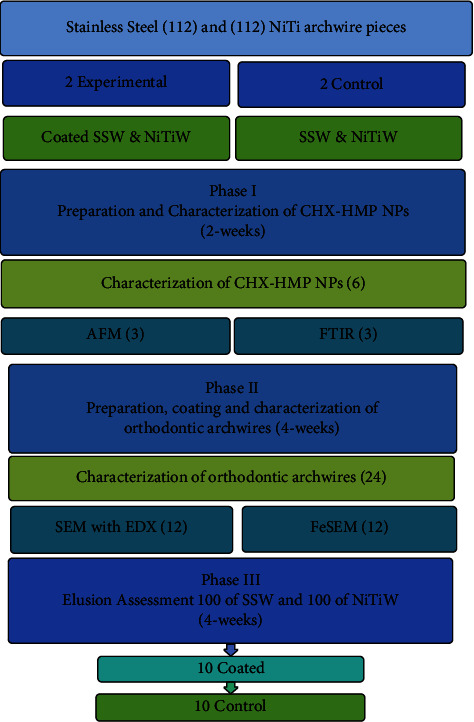
Diagram showing the study design, sample size, and timeline.

The complete timeline of this experimental study was 10 weeks. The total sample size involved was 224 pieces of orthodontic archwires. The study sample of archwire pieces was divided into four groups: two experimental groups of SSW and NiTiW coated with the CHX-HMP NPs, and the other two groups were noncoated control groups. This experimental study was divided into three phases (Figure 1). Phase I involved the preparation and characterization of coating material CHX-HMP NPs, and this step took two weeks and six samples of prepared nanomaterials were tested, 3 for atomic force microscope (AFM), and 3 for Fourier transformation infrared spectroscopy (FTIR). Phase II included the preparation, coating, and then characterization of orthodontic archwires, and this phase was performed in 4 weeks and involved the coating of 112 archwire pieces (56 SSW and 56 NiTiW) and the preparation of 112 control pieces (56 SSW and 56 NiTiW). Following that, 24 pieces of coated and control groups (12 SSW and 12 NiTiW) were characterized utilizing SEM with EDX (6 SSW and 6 NiTiW) and FeSEM (6 SSW and 6 NiTiW). Phase III focused on CHX elusion assessment, performed in 4 weeks, and involve 200 archwire pieces (100 SSW and 100 NiTiW). The sample size was determined in each subgroups (coated and control groups for SSW and NiTiW for each time interval) in the elusion test in accordance with ISO 15841 [31], which recommends ten wires per group.

### 2.3. Phase I: Synthesis and Characterization of CHX-HMP NPs

Under constant stirring, 100 mL of 10 mM aqueous sodium HMP was mixed with 100 mL of 10 mM aqueous CHX digluconate at room temperature and ambient conditions. This produced a 5 mM solution of CHX-HMP nanoparticles. By combining the two reagents, a colloidal suspension was produced instantaneously [26, 27]. The particle size of NPs in colloidal solutions was studied as a function of time using AFM (AFM workshop/TT-2, USA) and FTIR (IRPrestige-21, Shimadzu, Japan). Six samples were used for characterization; three for each test.

### 2.4. Phase II: Specimen Preparation, Coating, and Characterization

Straight segments of 30 mm length of orthodontic stainless steel and NiTi archwires were cut to be utilized in this study. The study sample of archwire pieces was divided into four groups: two experimental groups of SSW and NiTiW coated with the CHX-HMP NPs and the other two groups were noncoated control wires. The number of samples for each of these groups was 56 pieces.

All archwires of both experimental and control groups were cleaned to remove any contaminations by ultrasonication in deionized water for 15 min and then air-dried and sterilized utilizing ultraviolet (UV) light (Scie-plas GLE-UVSC UV sterilization cabinet, England) for 30 minutes [32]. To coat the sample, each sterilized archwire of the experimental groups was suspended in 10 ml of the previously prepared nanoparticles colloidal suspension by placing it in a test tube for a period of 30 seconds under rapid stirring using a rapid stirrer device (Nanolab-Scientific, Germany). After that, the sample was grasped carefully with a sterilized tweezer and immersed in deionized water for 10 sec to exclude any unbounded material and left to dry completely for at least 1 hour. These procedures were done in a biosafety cabinet. The sample, from each group (experimental and control), was kept in a sealed and clearly labeled test tube till the test day.

Characterization of the study sample, including both experimental and control groups, was done using SEM (FEI INSPECT S50, Czech Republic) and FeSEM (FEI INSPECT F50, Czech Republic) as well. ImageJ software program was employed to measure the size of nanoparticles on the coated archwire surface.

### 2.5. Phase III

#### 2.5.1. Preparation of the Artificial Saliva

Artificial saliva was prepared for the purpose of the elusion assessment. Modified Carter's solution formula was utilized including the following components and concentrations: 1.2 g KCl, 1.5 g NaHCO_3_, 0.26 g Na_2_HPO_4_, 0.7 g NaCl, 0.33 g KSCN, 0.2 g K_2_HPO_4_, and 0.13 g urea. All these ingredients were weighed using an electronic balance and then dissolved in 1000 ml of deionized water using a glass cylinder. For mixing the components, a glass rod was used till complete dissolution in water. After that, the mixture was filtered using 0.5 *μ*m pore filter paper to exclude any impurities to make the saliva as pure as possible. In the next step, sodium hydroxide (NaOH) and lactic acid were added for adjusting the pH value of the artificial saliva to 6.75 ± 0.15. The pH value was measured using a pH meter (Lovibond SD 300 PH, China) [33, 34].

#### 2.5.2. Assessment of CHX Elusion the Coated Archwires

The concentrations of the released soluble CHX from the CHX-HMP NPs-coated orthodontic archwire segments were measured for a period of 28 days. For this assessment, SSW and NiTiW (*n* = 200) for each of them (10 coated, 10 control from each type for every experimental period) were used. The sample size was determined in accordance with ISO 15841 [31], which recommends ten wires per group.

Each archwire segment from the experimental and control groups of both SSW and NiTiW was immersed in a test tube containing 5 mL of artificial saliva and sealed with a secure lid, and then the joint was protected with parafilm. In the next step, the sample was placed in an orbital shaker at 37°C and 150 rpm. After the predetermined period for each sample was completed (at 1 day, 7 days, 14 days, 21 days, and 28 days), CHX concentrations were subsequently measured by registering the absorbance at 255 nm utilizing a UV spectrophotometer (UV-1900i, Shimadzu, Kyoto, Japan). For the calibration of CHX concentrations, a standard solution of 5–50 *µ*M of CHXdg was carried out as a reference [26, 27, 35].

### 2.6. Statistical Analyses

SPSS version 26 was used for all statistical analyses. Minimum, maximum, mean, standard deviation, standard error, and Shapiro–Wilk test were all utilized to characterize the data. A 95% confidence interval is typically used in inferential statistics. When the *p* value was less than 0.05, it was determined to be statistically significant. An independent *t*-test was used for comparing the release difference between stainless steel and NiTi orthodontic archwires.

## 3. Result

### 3.1. Characterization of Nanoparticles

#### 3.1.1. AFM

**Figure 2:**
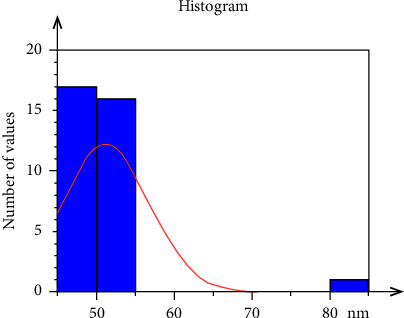
Histogram of the nanoparticles in the colloidal suspension explaining the nanoparticles' size and distribution.

**Figure 3:**
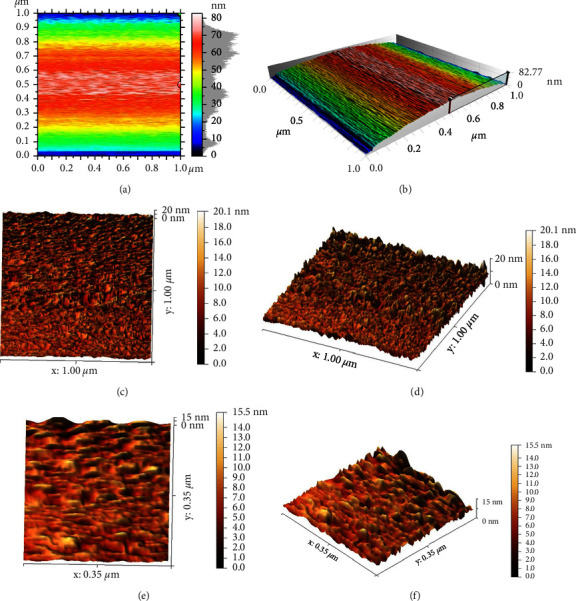
AFM topographical micrograph for the prepared CHX-HMP nanoparticles: (a) 2D at 1 *μ*m, (b–d) 3D at 1 *μ*m, and (e, f) at 0.35 *μ*m.

Generally, the nanoparticles analysis by AFM showed that the prepared material had a nanoscale size, as the histogram (Figure 2) demonstrated the nanoparticles size distribution which ranged between 49.3 and 82.7 nm with an average of 51.2 nm (Table 1). The AFM micrographs explained that the nanoparticles were characterized by relative homogeneity in terms of particle size and in their topographical distribution. They also showed that the particles have regular shapes and are well aligned vertically as seen in the 2D and 3D AFM micrographs (Figure 3).

#### 3.1.2. FTIR

The FTIR spectrum chart of chlorhexidine digluconate liquid of the nanoparticles prior to mixing explained the presence of characteristic transmittance bands for the N-H group at 3317 cm^−1^, 3197 cm^−1^ for the O-H group, and 2935 cm^−1^ for C-H of the methanediyls group. Other bands were found at 1630 cm^−1^ for C=O, 1600 for N-H bending, 1539 cm^−1^ for (C=N), and 1415 cm^−1^ for (C=C aromatic). Moreover, 1369 cm^−1^ (bending vibration of C-H methanediyl), 823 cm^−1^ (C-H bending vibration of C-H aromatic), and 715 cm^−1^ (C-Cl aromatic) were found.

While for sodium hexametaphosphate crystalline powder, the FTIR spectrum showed peaks at 1280 cm^−1^ for the P=O, the vibration of P-O appeared at 1105 cm^−1^, and the vibration at 883 cm^−1^ was due to P-O-P.

However, for chlorhexidine hexametaphosphate colloidal suspension of the prepared nanoparticle, FTIR spectrum, in which the spectra were measured at a range between 400 and 4000 cm^−1^ in the transmittance mode, showed broad peaks at 3437 cm^−1^ mostly referring to the N-H bond, whereas at 3060 cm^−1^, a small band for stretching vibration of hydrogen of phenol ring was seen. The small band or peak at 2950 cm^−1^ is due to the stretching vibration of C-H of CH2. While for the aromatic guanidine absorptions, peaks were found at 1699 cm^−1^ for the ArNHC(=NH) NHAr), 1606 cm^−1^ for C=N and for the C=C aromatic, 1269 cm^−1^ for the P=O, 932 cm^−1^ for the P-O-P, and 516 cm^−1^ for the C-Cl aromatic groups and 429 cm^−1^ for bending vibration of C-H of the phenol ring.

**Figure 4:**
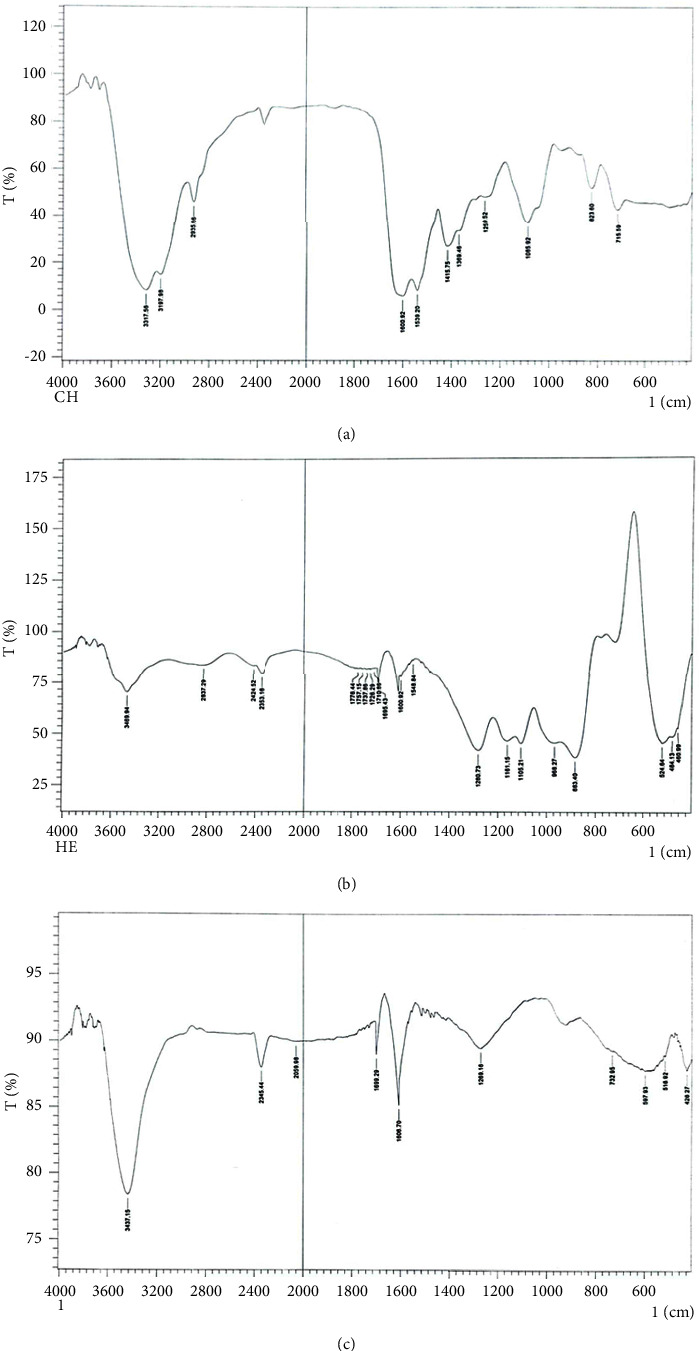
FTIR spectra charts in the range of 400–4000 cm^−1^ in KBr pellet. (a) Chlorhexidine digluconate liquid. (b) Sodium HMP crystalline powder. (c) Chlorhexidine HMP colloidal suspension nanoparticles after mixing.

Furthermore, in the FTIR charts, there were specific characteristic bands that indicate the chemical interaction between CHX digluconate and sodium hexametaphosphate. The presence of a strong band of C=O in chlorhexidine digluconate and its absence in the mixed nanoparticles indicate the replacement of gluconate by hexametaphosphate. In addition to that, the presence of peaks of phosphate such as P=O, P-O, and P-O-P and the presence of Cl groups in the resultant colloidal suspension of the prepared nanoparticles may indicate the interaction between the sodium hexametaphosphate and chlorhexidine digluconate after mixing (Figure 4).

### 3.2. Characterization of Orthodontic Archwires

#### 3.2.1. SEM

**Figure 5:**
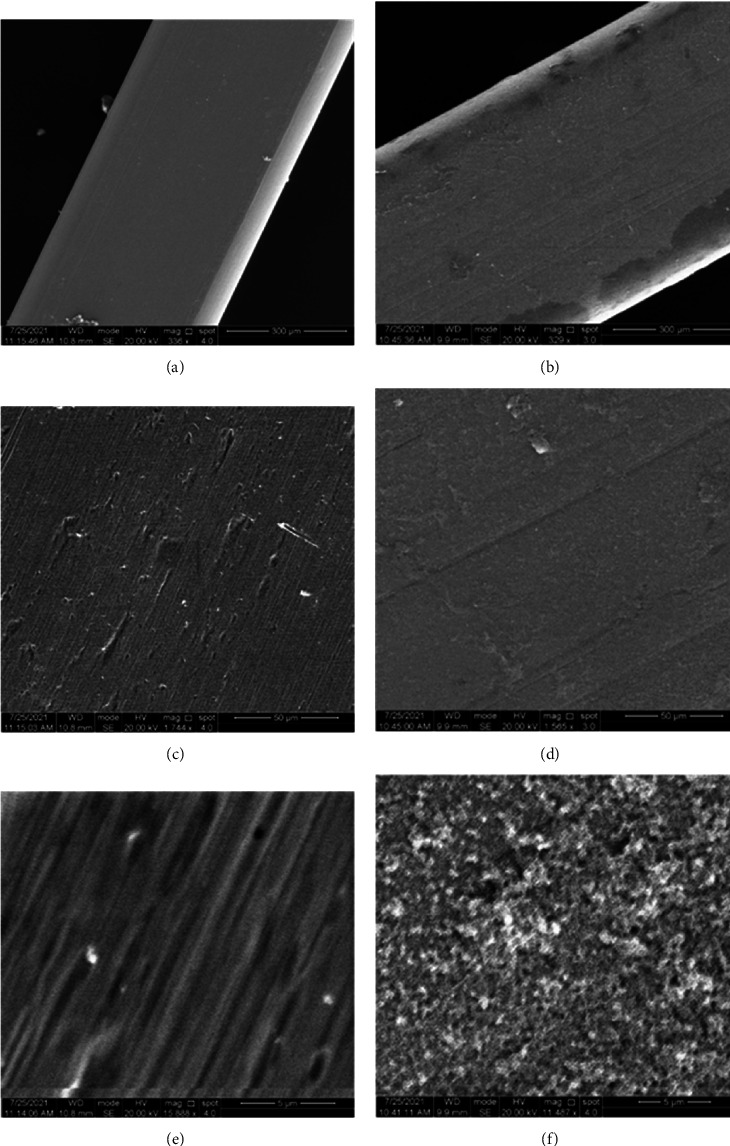
SEM images for the examined SSW at different magnification powers. (a, c, e) uncoated and (b, d, f) coated.

**Figure 6:**
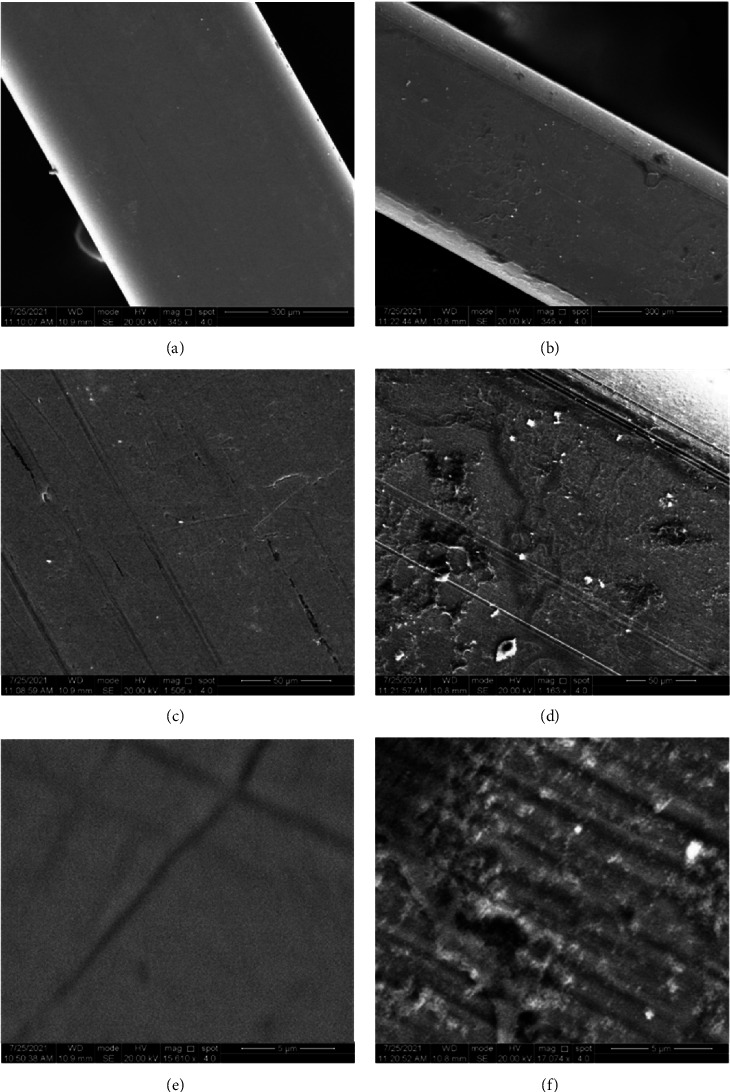
SEM images for the examined NiTiW at different magnification powers. (a, c, e) uncoated and (b, d, f) coated.

SEM images at different magnification powers showed the difference between the surface topography of both SSW and NiTiW uncoated control archwire segments and the coated ones with CHX-HMP NPs for both tested materials (Figures 5 and 6, respectively). SEM images for the coated SSW at different magnifications demonstrated more homogeneity and almost equal distribution of the prepared nanoparticles.

#### 3.2.2. Characterization by EDX Spectroscopy

**Figure 7:**
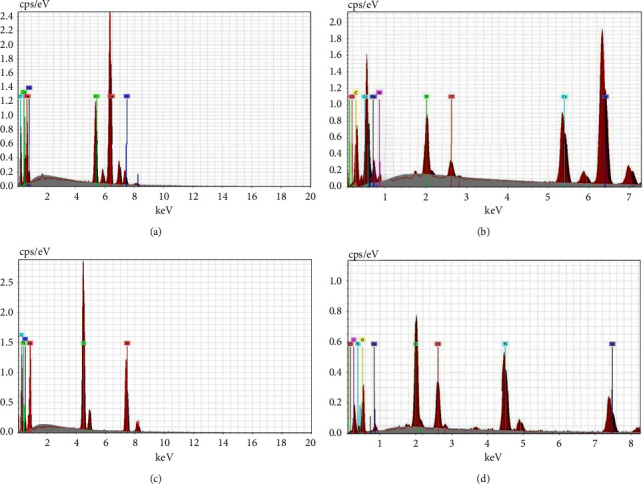
EDX charts for the examined SSW: ((a) before coating and (b) after coating) and the examined NiTiW ((c) before coating and (d) after coating).

Micrographs of EDX characterization showed the chemical elemental analysis and ionic distribution in all sample groups of control and coated SSW and NiTiW with CHX-HMP NPs in Figure 7. EDX analysis demonstrated the presence of chlorine (Cl) and phosphorus (P) atoms on the coated orthodontic archwires segments of both SSW and NiTiW groups when compared with the control groups and reduce the levels of the essential elements in both materials. This clearly reveals the covering of the specimens with the CHX-HMP NPs.

#### 3.2.3. Characterization by FeSEM

**Figure 8:**
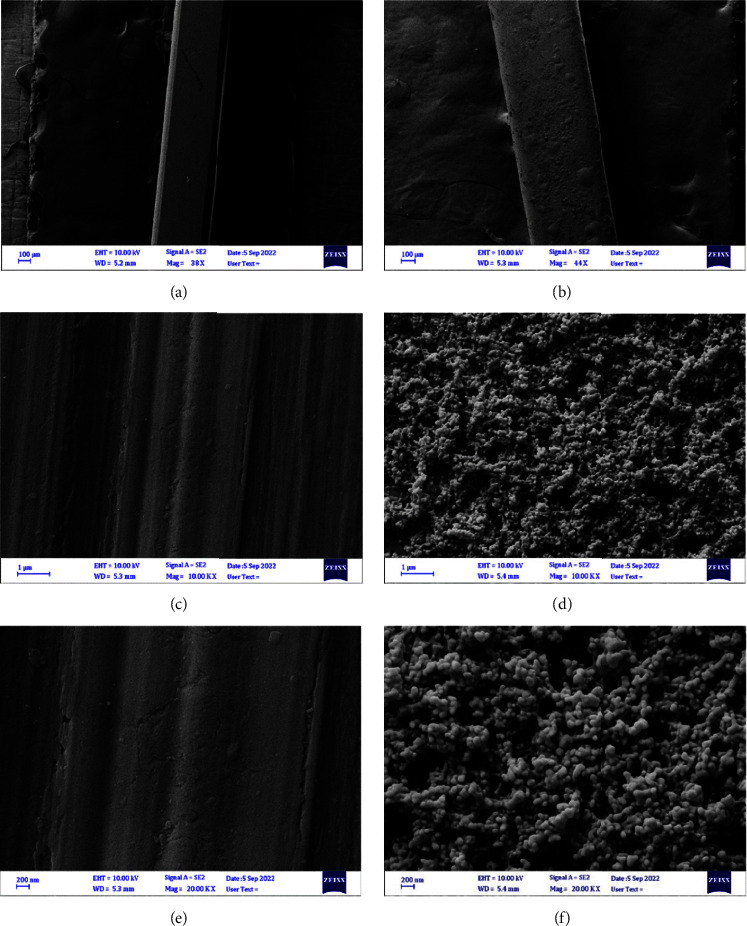
FeSEM images for the examined SSW at different magnification powers: (a, c, e) uncoated and (b, d, f) coated.

**Figure 9:**
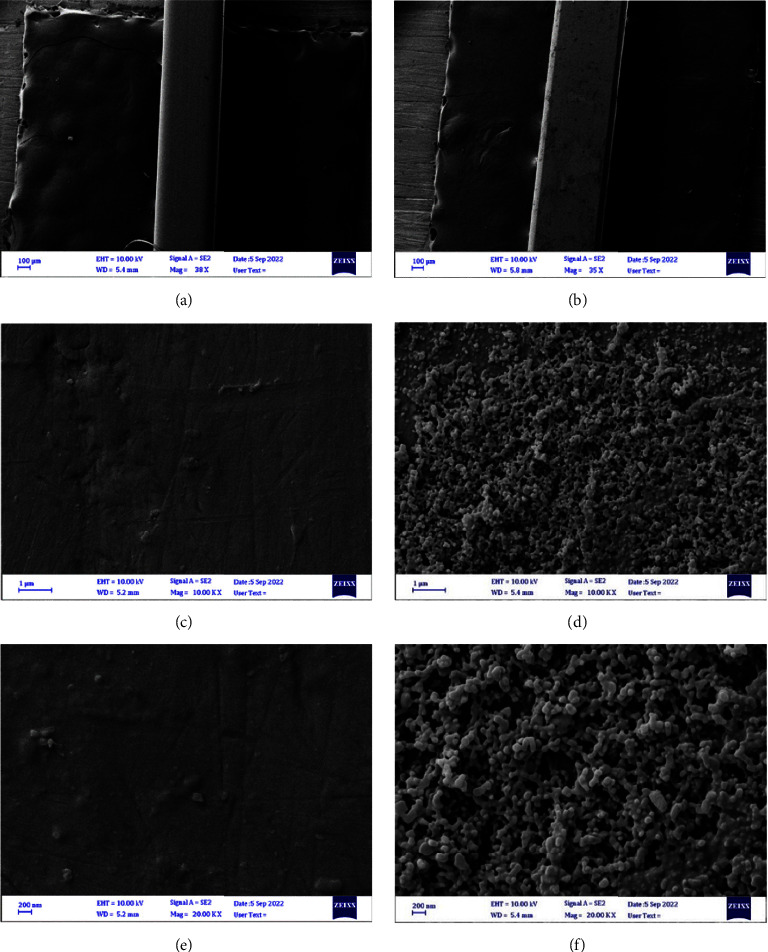
FeSEM images for the examined NiTiW at different magnification powers: (a, c, e) uncoated and (b, d, f) coated.

**Figure 10:**
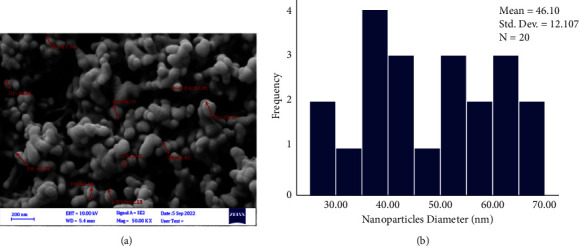
Size estimation of the nanoparticles using the FeSEM images and ImageJ software on the coated SSW. (a) Nanoparticles measurement. (b) Size distribution histogram of the nanoparticles.

FeSEM images at various magnifications indicated the difference between the surface topography of the uncoated control archwire segment in comparison to coated ones with CHX-HMP NPs for both SSW and NiTiW groups (Figures 8 and 9, respectively). The images of the CHX-HMP nanoparticles coated SSW and NiTiW segments showed that the nanoparticles had a spherical shape with a normal and homogenous distribution. Additionally, the absence of agglomerations in the images indicated that the produced colloidal suspension had effectively dispersed nanoparticles onto the surfaces of the samples. ImageJ software determined that the average particle size of the coated SSW for 20 particles was 46.1 nm and that the particle size ranged from 27.2 to 67.3 nm (Figure 10).

**Figure 11:**
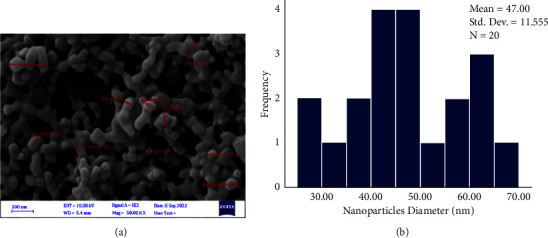
Size estimation of the nanoparticles using the FeSEM images and ImageJ software on the coated NiTiW. (a) Nanoparticles measurement. (b) Size distribution histogram of the nanoparticles.

Measuring the particle size on coated NiTiW using ImageJ software for 20 particles showed that the average particle size was 47 nm, with a range of 26–67.13 nm (Figure 11).

### 3.3. CHX Elusion from CHX-HMP NPs-Coated Archwires

**Figure 12:**
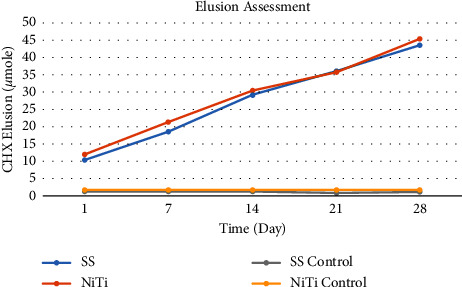
Elusion of accumulative CHX from coated SSW and NiTiW along 28 days compared with the control specimens.

Throughout the course of the experiment, CHX-HMP NPs-coated orthodontic archwire segments of both stainless steel and NiTi continuously released soluble CHX. Nanoparticle-coated SSW released 10.24 *μ*mole of CHX on day one while coated NiTiW released about 11.89 *μ*mole. Subsequently, the release measurements from coated SSW were 18.49, 29.23, 36, and 43.48 *μ*mole after 7, 14, 21, and 28 days, respectively. At the same time, CHX release values from coated NiTiW ranged from 19.75 to 31.48 to 36.6 to 46.9 *μ*mole over the course of the experiments. Release from coated NiTiW was more than that from SSW, as demonstrated in the preceding data (Figure 12).

According to an independent *t*-test, the release of CHX from coated NiTiW was significantly more than that of SSW for the first, 7, 14, and 28 days, while for the 21 days period, the release from coated SSW was slightly greater than NiTiW (Table 2).

The raw data of the examined samples for SSW are shown in Table 3 and for NiTiW in Table 4.

## 4. Discussion

The application of nanoparticles as antimicrobial agents is gaining a great importance in dentistry as it may provide an appreciated strategy for treating and preventing dental infections [36]. There are two main approaches to incorporating the nanoparticles with antimicrobial effects in orthodontics, either by combining them directly with the materials such as composites, glass ionomers, or topically applied agents [37, 38] or utilize them as coatings on the surfaces [39].

Orthodontic stainless steel and NiTi archwires are used fundamentally during the course of orthodontic treatment with fixed appliances and are considered a common site for the development of microbial plaque [40, 41]. Several studies evaluate the microbial adhesions to different orthodontic archwires following intraoral wearing of the appliances that concluded that biofilm adherence increased in the archwires with time and the surface roughness of archwires was positively correlated with biofilm adherence [42–44].

Although the idea of localized antibiotic administration is not new, clinical and laboratory trials have produced somewhat conflicting outcomes. This may be attributed to the difference in the delivery systems [45]. Many studies attempt to coat orthodontic archwires with different antimicrobial agents trying to reduce oral microbiomes like photocatalytic TiO_2_ [46], silver material to coat stainless steel and NiTi archwires [47], N-doped TiO_2_ to coat composite, NiTi and SS wires [48], and silver nanoparticles to coat stainless steel archwires [49]. The closeness of orthodontic archwires to the enamel surface makes them the most suited and practical vehicle for the delivery of antimicrobial compounds where they are most needed. Since the archwires are constantly being changed during treatment, this will provide a fresh source of antimicrobial agents [47].

In this investigation, CHX-HMP NPs were selected as a coating material since the previous research studies have demonstrated that these nanoparticles can readily be adsorbed into the specimen surface, formed adherent nanoparticles, and extended the time that CHX was released [26, 27, 30]. The manufacturer's instructions, which were followed in the earlier investigations [26–29], were also followed in this work to create the colloidal suspension of nanoparticles.

This study selected the 0.019 × 0.025-inch stainless steel and NiTi orthodontic upper archwires only as a representative sample of different archwires sizes and cross sections.

AFM was used to examine the particle shape, distribution, and size of the produced colloidal suspension. This is due to the fact that it is one of the most successful and proven approaches for characterization, particularly for nanoparticles with diameters less than 50 nm [50]. The average size of CHX-HMP nanoparticles in the prepared suspension was found to be 51.21 nm; they were well-distributed, homogenous, and had regular forms.

An infrared (IR) spectrum is the fingerprint of the material, with absorption peaks corresponding to the frequencies of atomic bond vibrations [51]. Due to the fact that each material has a unique composition and, consequently, a different atomic arrangement, no two compounds create the same IR spectrum. Consequently, IR spectroscopy can positively identify (qualitatively analyze) every type of material. In addition, the magnitude of a spectrum's peak is directly proportional to the amount of material present. Using modern software methods, infrared (IR) is a great quantitative analysis tool [52]. Therefore, to determine the nature of the chemical bond between the chlorhexidine and hexametaphosphate nanoparticles in the produced colloidal suspension, FTIR spectroscopy was used. Besides the presence of phosphate and Cl groups in the suspension, the absence of C=O that exists in the chlorhexidine digluconate indicates that gluconate has been replaced by hexametaphosphate.

The SEM is considered one of the best-certified tools for providing direct images of coated and uncoated surfaces used to examine nanoparticles' topography and shape of the sample. EDX spectroscopy is usually utilized to discover the essential elemental composition of a substance [53]. As observed in the SEM and FeSEM images, the surfaces of the nanoparticle-coated archwires of both groups showed that the nanoparticles were evenly and uniformly distributed.

The average nanoparticles size of the coated SSW for 20 particles was 46.1 nm and for coated NiTiW was 47 nm, as calculated using ImageJ software. The slight difference between the average of nanoparticles in the suspension and on the coated samples may be attributed to the time role as the coating procedure was done as a function of time to avoid nanoparticles agglomerations.

The rapid attachment of the CHX-HMP NPs to the stainless steel and NiTi surfaces is thought to be the result of electrostatic attraction caused by the particles' highly charged nature; this is substantiated by the successful deposition of the same nanoparticles on diverse surfaces, including glass and ethylene vinyl acetate (EVA), a commonly used biomedical polymer [26, 27, 30, 54].

In this study, artificial saliva was selected to be used in the elusion assessment test instead to mimic the intraoral scenario, while all previous studies were done on CHX-HMP NPs-coated biomaterials utilizing deionized water. Based on the findings of this investigation, the cumulative concentration of chlorhexidine emitted from both stainless steel and NiTi archwire segments coated with CHX-HMP NPs grew steadily over time. This identifies that the addition of HMP nanoparticles induced a gradual and continuous release of CHX, leading to prolonging the antimicrobial effect. This supports the evidence that HMP nanoparticle can act as an active transporter for CHX and allow sustained slow release [26, 27, 30].

CHX digluconate salt is utilized in commercial CHX products such as rinses, gels, sprays, toothpaste, lozenges, and varnishes because it is extremely soluble in water [55]. Due to the solubility of the CHX salt, which is immediately eluted upon contact with fluid, it results in a high initial concentration and no continuous release [26], limiting its application as a biomaterial coating.

The result of the study showed that CHX was initially released as a burst on the first day and then at a nearly continuous rate as long as the experiment lasted for 28 days without reaching a plateau, and this may indicate continuous release for a longer time as reported by Barbour et al., 2013, that found that CHX-HMP NPs slow down the release of CHX. When glass or titanium specimens are coated with the same nanomaterial, aqueous CHX keeps leaking out for at least 56 days without stopping [26]. Also, Kamarudin et al. studied with coated orthodontic elastomeric ligatures and found that CHX-HMP continued to release CHX even after 8 weeks of contact with an aqueous environment [28].

For the first, seventh, fourteen, and twenty-eighth days, CHX release from coated NiTiW was significantly more than SSW, whereas for the twenty-one days, release from coated SSW was somewhat greater than NiTiW. These results could be explained as due to the greater surface roughness of NiTi archwires than that of SSW [56], which may lead to more amounts of CHX-HMP NPs accumulation within the pores or voids on the NiTiW surface.

### 4.1. Limitations of the Study

This is an experimental (in vitro) study; therefore, even with the best efforts to simulate the oral conditions by using artificial saliva and an orbital shaker, it is still different from the influence of the intraoral scenario on the surface of the coated materials.

### 4.2. Strength of the Study

This study can be seen as the first study that investigates the coating of orthodontic archwires with CHX-HMP NPs and assesses CHX elusion. Furthermore, the relatively simple coating procedure, low cost of coating materials, and chlorhexidine are considered a gold standard antimicrobial agent, and all this encourages the employment of CHX-HMP NPs as a promising coating material for orthodontic archwires.

### 4.3. Clinical Implications

The appropriate coating of archwires and releasing of the CHX candidate is to be assessed in a clinical environment in an attempt to reduce the occurrence of enamel demineralization and gingival and periodontal problems.

## 5. Conclusion

The surfaces of stainless steel and NiTi orthodontic archwires can be coated with CHX-HMP NPs. Over the course of the experiment, this coating steadily released soluble CHX.

## Figures and Tables

**Table 1:** The AFM grain statistics for the prepared CHX-HMP nanoparticles.

Number of particles	425
Coverage	58.3%
Density	34000000 particles/mm^2^
Mean size of the particles	51.21 nm
Minimum size of the particles	49.35 nm
Maximum size of the particles	82.77 nm
SD	5.562
Mean of projected area	0.01728 *µ*m^2^
Root mean square height (*Sq*)	10.54 nm
Maximum height (*Sz*)	59.09 nm
Arithmetic mean height (*Sa*)	8.116 nm

**Table 2:** Comparison of the CHX elusion (*μ*mole) of coated SSW and NiTiW.

Duration	Groups	*N*	Min	Max	Mean	SD	SE	Shapiro–Wilk test (*p* value)	Independent samples *t*-test (*p* value)
1 day	SSW	10	8.50	13.15	10.24	1.51	0.48	0.265	**0.02**
	NiTiW	10	10.12	14.23	11.89	1.36	0.43	0.691	

7 days	SSW	10	16.86	21.10	18.49	1.32	0.41	0.425	**0.001**
	NiTiW	10	17.97	23.58	21.20	1.83	0.57	0.529	

14 days	SSW	10	27.14	31.59	29.23	1.33	0.42	0.961	**0.034**
	NiTiW	10	28.72	32.00	30.50	1.14	0.36	0.648	

21 days	SSW	10	33.89	37.43	36.00	1.12	0.35	0.623	0.471
	NiTiW	10	33.50	37.00	35.60	1.26	0.40	0.328	

28 days	SSW	10	40.75	45.86	43.48	1.78	0.56	0.561	**0.007**
	NiTiW	10	44.00	47.00	45.43	0.98	0.31	0.826	

The mean difference is significant at the 0.05 level.

**Table 3:** The raw data of CHX elusion of SSW.

Elusion of CHX from SSW									
Coated 1 day	Control 1 day	Coated 7 days	Control 7 days	Coated 14 days	Control 14 days	Coated 21 days	Control 21 days	Coated 28 days	Control 28 days
9.1	0.8	18.5	1.6	29.83	1.5	37.23	0.9	40.75	1.6
10.5	1.7	20.16	1	28.35	1	36.42	1	44.34	1
12.25	1	18.25	0.88	29.65	0.95	35.44	1	43.5	0.8
8.5	0.8	17.15	0	27.85	0.7	35.95	0.5	45.86	2
9.15	0.5	18.11	0.98	30.26	0	35.9	0.8	42.64	0.98
11	0	16.86	1.6	28.9	0.5	34.75	0.9	41.85	1.6
9.5	1.5	17.5	0.9	28.48	1.9	33.89	1	43	0.9
9	1	21.1	0.95	27.14	1.8	35.87	1	45.32	0.95
13.15	0.95	18.13	1	31.59	1.6	37.43	0.6	41.83	1.8
10.25	0.99	19.14	0.89	30.25	1.7	37.12	1.8	45.71	0.9

**Table 4:** The raw data of CHX elusion of NiTiW.

Elusion of CHX from NiTiW									
Coated 1 day	Control 1 day	Coated 7 days	Control 7 days	Coated 14 days	Control 14 days	Coated 21 days	Control 21 days	Coated 28 days	Control 28 days
10.73	1.9	23.58	1.5	31.95	2	33.5	2	44.15	2.5
12.48	2	22.36	1	30.16	1.4	35	1.4	47	1.9
11.31	0.8	20.85	0.95	29.67	1.8	36.8	1.8	46.15	1.8
14.23	2	19.89	0.7	30.9	1.8	37	2	45.41	2
12.29	1.5	18.72	2	31.4	0.98	35.9	0.98	45	0.98
10.12	1	17.97	1	29.23	1.6	34.75	1.6	45.92	1.6
12.66	0.9	21.84	1.9	28.72	1.7	34	1.9	46.35	1.9
11.15	0.9	22.52	2	29.85	1	37	2	45.76	2.2
13.52	1.8	22.79	1.6	32	1.8	35.5	1.8	44.56	1.8
10.41	0.9	21.48	1.7	31.12	1.5	36.6	1.6	44	1.7

## Data Availability

The data used to support the findings of this study are included within this article
